# Birds of a feather flock together: a dataset for *Clock* and *Adcyap1* genes from migration genetics studies

**DOI:** 10.1038/s41597-023-02717-8

**Published:** 2023-11-09

**Authors:** Louis-Stéphane Le Clercq, Gaia Bazzi, Joan Ferrer Obiol, Jacopo G. Cecere, Luca Gianfranceschi, J. Paul Grobler, Antoinette Kotzé, Marta Riutort León, Jacob González-Solís, Diego Rubolini, Miriam Liedvogel, Desiré Lee Dalton

**Affiliations:** 1 South African National Biodiversity Institute, P.O. Box 754, Pretoria, 0001 South Africa; 2 Department of Genetics, University of the Free State, P.O. Box 339, Bloemfontein, 9300 South Africa; 3 Area Avifauna Migratrice, Istituto Superiore per la Protezione e la Ricerca Ambientale, via Ca’ Fornacetta 9, I-40064 Ozzano Emilia, BO Italy; 4 Departament de Genètica, Universitat de Barcelona, Gran Via de les Corts Catalanes, 585, 08007 Barcelona, Spain; 5 Institut de Recerca de la Biodiversitat (IRBio), Universitat de Barcelona, Gran Via de les Corts Catalanes, 585, 08007 Barcelona, Spain; 6 Dipartimento di Scienze e Politiche Ambientali, Università degli Studi di Milano, via Celoria 26, Milan, I-20133 Italy; 7 Dipartimento di Bioscienze, Università degli Studi di Milano, via Celoria 26, Milan, I-20133 Italy; 8 Departament de Biologia Evolutiva, Universitat de Barcelona, Gran Via de les Corts Catalanes, 585, 08007 Barcelona, Spain; 9 Istituto di Ricerca sulle Acque, IRSA-CNR, Via del Mulino 19, I-20861 Brugherio, (MB) Italy; 10 Max Planck Research Group Behavioural Genomics, Max Planck Institute for Evolutionary Biology, 24306 Plön, Germany; 11 Institute of Avian Research, An der Vogelwarte 21, 26386 Wilhelmshaven, Germany; 12 School of Health and Life Sciences, Teesside University, Middlesbrough, TS1 3BA UK

**Keywords:** Behavioural genetics, Animal migration, Animal migration

## Abstract

Birds in seasonal habitats rely on intricate strategies for optimal timing of migrations. This is governed by environmental cues, including photoperiod. Genetic factors affecting intrinsic timekeeping mechanisms, such as circadian clock genes, have been explored, yielding inconsistent findings with potential lineage-dependency. To clarify this evidence, a systematic review and phylogenetic reanalysis was done. This descriptor outlines the methodology for sourcing, screening, and processing relevant literature and data. PRISMA guidelines were followed, ultimately including 66 studies, with 34 focusing on candidate genes at the genotype-phenotype interface. Studies were clustered using bibliographic coupling and citation network analysis, alongside scientometric analyses by publication year and location. Data was retrieved for allele data from databases, article supplements, and direct author communications. The dataset, version 1.0.2, encompasses data from 52 species, with 46 species for the *Clock* gene and 43 for the *Adcyap1* gene. This dataset, featuring data from over 8000 birds, constitutes the most extensive cross-species collection for these candidate genes, used in studies investigating gene polymorphisms and seasonal bird migration.

## Background & Summary

Birds occupy nearly every habitat and ecoregion on Earth, however, many of these habitats experience large seasonal shifts in key ecological attributes such as length of day^1^, temperature^2^, rainfall^3,4^, and associated food and nesting material availability^5^. This has necessitated the adaptive evolution of complex strategies to maximise survival through seasonal migrations between breeding and wintering ranges. Migrations are carefully timed events, scheduled in such a manner that birds can optimise hours of daylight^6^, nighttime visibility^7,8^, and time spent at stop-over sites^9^ along their migration route to ensure timely arrivals for optimal habitat use. While most of the ecological attributes play some role in the timing of migration, one of the best studied attributes that serve as a trigger to initiate migration is the length of day or photoperiod. The photoperiod is primarily responsible for daily oscillations within the regulatory feedback loops of the circadian clock, which differentially expresses genes during light or dark phases to maintain sleep-wake cycles in most organisms^10^.

One conundrum regarding migration in birds is how differential migration patterns are established and maintained within singular species, even in the absence of extrinsic environmental triggers. For example, several species within the order Coraciiformes have distinct populations that are either year-round residents, with minimal altitudinal movement, or long-distance migrants. This includes such species as the Lilac-breasted roller^11^ (*Coracias caudatus*) and Woodland kingfisher^12^ (*Halcyon senegalensis*), both having subspecies that are delineated by differential migration, as well as the European bee-eater^13^ (*Merops apiaster*), which is considered monotypic but has a distinct resident population in Southern Africa. Understanding how differential migration is established and maintained between such species is key to assessing connectivity^14^, speciation at a subspecies level^15^, and potential population fitness^16^. This is particularly pertinent with regards to the plasticity or ability to switch between behaviours^17,18^ should environmental conditions change considerably due to climate change^19–21^ or anthropogenic activity^22–25^.

Several studies have explored the possible genetic components that affect intrinsic time keeping mechanisms and migration. Although variable methods have been used, including genomic^26^, epigenetic^27^, and transcriptomic approaches^28^, most studies sought to identify genes or gene regions that show variation in either the sequence itself or the gene expression that can be correlated to divergent migratory behaviour. The key, however, is identifying variation that is linked to processes that interface with annual life events. Thus, variation that is either connected to the endocrine or metabolic changes^29^, in preparation for migration and breeding, or intrinsic time-keeping mechanisms, such as the rhythmic expression of circadian genes; particularly those that interface with environmental changes that my serve as cues such as photoperiod, temperature, lunar cycles, and food availability^30^. This is needed to exclude variants that co-vary with migration phenotypes but are not actively involved in shaping them. It is therefore no surprise that many candidate gene studies have explored variation within the network of genes of the circadian clock. Several associated candidate genes have been suggested, with length polymorphisms within short repeats of the *Clock* and *Adcyap1* genes being the focus of many studies^31–33^.

**Fig. 1:**
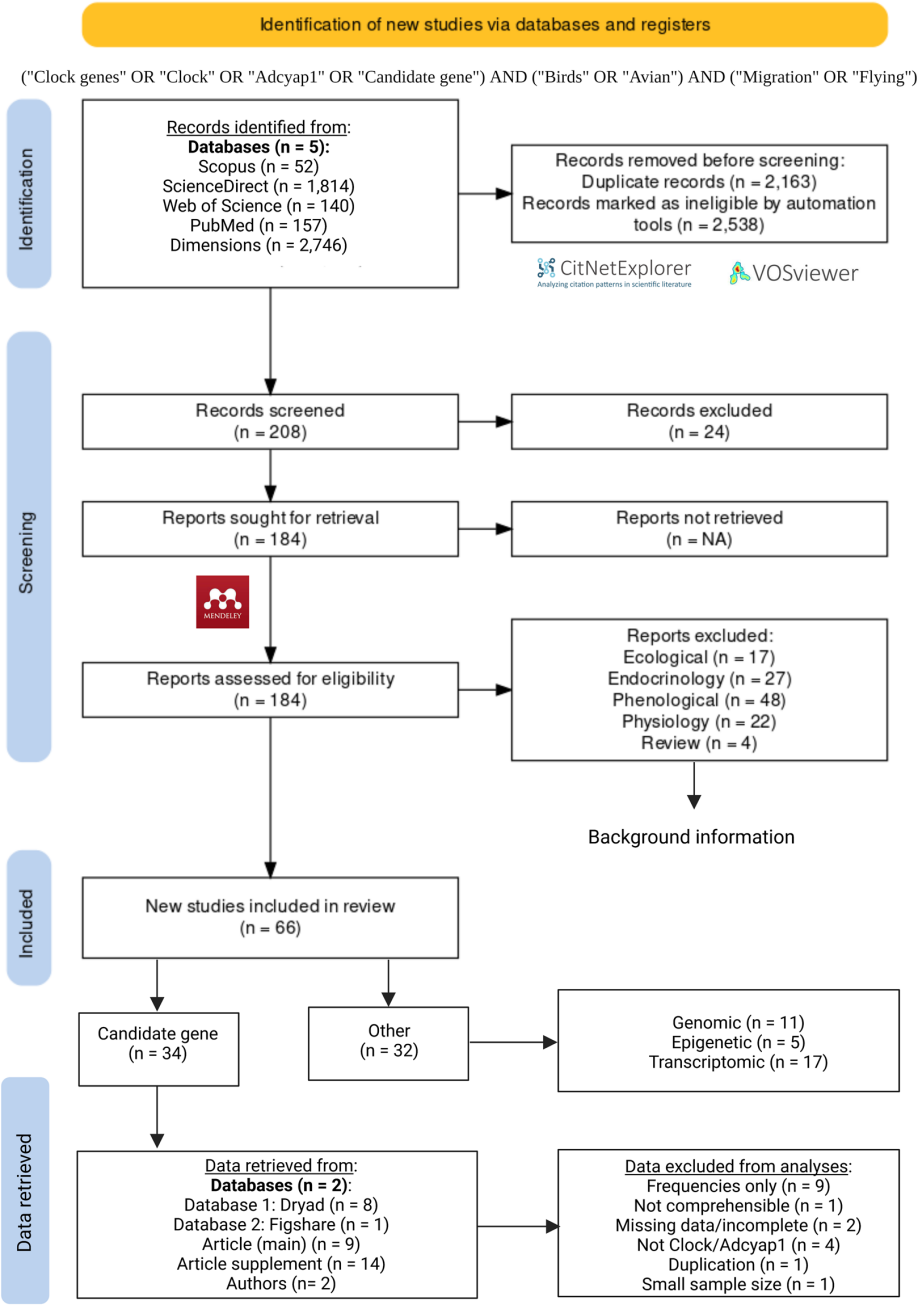
PRISMA statement for the systematic approach used to identify studies that measured clock gene polymorphisms in relation to annual synchronicity of live events such as breeding and migration in birds. Further details are also provided for the retrieval of allele data for individual studies from various sources as well as reasons for exclusion of studies. (image edited in BioRender.com).

To clarify the role of these genes in migratory phenotypes, a systematic review (Fig. 1) was conducted to identify, synthesise, and provide a reappraisal of the available evidence^34^. Structured searches of the literature with an optimised Boolean search string were done in five scientific databases. Search results were exported in formats compatible with citation network analysis software^35^. After duplicate entries were removed, citation network analyses were used for the automated screening of database results to identify the central literature on the topic. Publications identified from the citation network analyses were subjected to manual screening of the title, abstract, and key words to assess the potential eligibility for inclusion in the review. The final list of most eligible publications was sought for full text retrieval. A total of 66 studies were included in the final review of which 34 were candidate gene studies and 32 were other, migration-related, studies. These included latitude/longitude/spatial analyses, timing of migration, and timing of egg laying/breeding. Most of the studies using a candidate gene approach were used for data retrieval. For these studies, datasets were retrieved as either diploid allele data of individuals or allele frequencies. Data sources included the main text of articles, supplementary materials, databases such as Dryad (https://datadryad.org/) or Figshare (https://figshare.com/), data extraction, or data received directly from authors. Unpublished data for an additional 12 species were also included. The dataset included individual level allele data from 52 species of which data was available for 46 species for the *Clock* gene and 43 species for the *Adcyap1* gene. This dataset represents the largest collection of cross species allele data for two candidate genes used to test a putative association between clock gene polymorphisms and divergent migration in birds, which enables the testing for patterns of inheritance, evolutionary selection, relation to divergence times, and associations across a globally distributed dataset.

This data descriptor summarises both the methodology used to screen the literature as well as to compile the data concisely and presents the resulting data used in prior analyses in an easy-to-understand format. At present, none of the scientific databases that collect genetic variation data is suitable for the deposit of this specific type of data. The barcode of life data system (BOLD, https://boldsystems.org/), which does accept length polymorphism data from microsatellite markers, currently only accepts data for markers used in barcoding or population assignment experiments and does not specifically store data for markers used in behavioural or phenotype associated studies. The European variant archive (EVA, https://www.ebi.ac.uk/eva/), which also accepts variant data that includes length polymorphisms, currently only accepts data for species with reference genomes, which is still unavailable for most avian species. To overcome this, we have endeavoured to create a central compilation of the available data in two standard formats which is archived in parallel to this data descriptor; with an additional online version on GitHub^36^ (https://github.com/LSLeClercq/AvianClocksData) that will be maintained and updated over time as more data is made available. This may greatly facilitate the reuse of the data where it may be applicable to other forms of analyses within migration genetics and beyond.

## Methods

### Literature search and automated screening

Literature was searched using systematic review methods, in line with PRISMA Ecology and Evolution guidelines^37^, to identify and synthesize relevant sources. The overall approach is depicted in the PRISMA statement^38^ in Fig. 1 that was supplemented with further information on the data retrieval and screening process. Literature was searched between January and September of 2022 on five databases: Scopus (N = 52, www.scopus.com), ScienceDirect (N = 1814, www.sciencedirect.com), Web of Science (N = 140, https://clarivate.com/), PubMed (N = 157, https://pubmed.ncbi.nlm.nih.gov/), and Dimensions (N = 2746, www.dimensions.ai). Databases were searched using an optimized Boolean search string derived from the PICO terms for the aim and objectives of the review. The final search string was as follows: (“Birds” OR “Avian”) AND (“Clock genes” OR “Clock” OR “Adcyap1” OR “Candidate gene”) AND (“Migration” OR “Flying”). As needed, this was complemented by ancillary ‘free term’ searches based on citations in articles or to include other relevant aspects such as “Breeding”, “Moult”, “Genomics”, “Transcriptomics” or “Photoperiod”. For the Scopus and Dimensions database searches, the results were exported in the comma separated value (CSV) format, while the results from the ScienceDirect, Web of Science, and PubMed database search were exported in the research information systems (RIS) format.

**Fig. 2:**
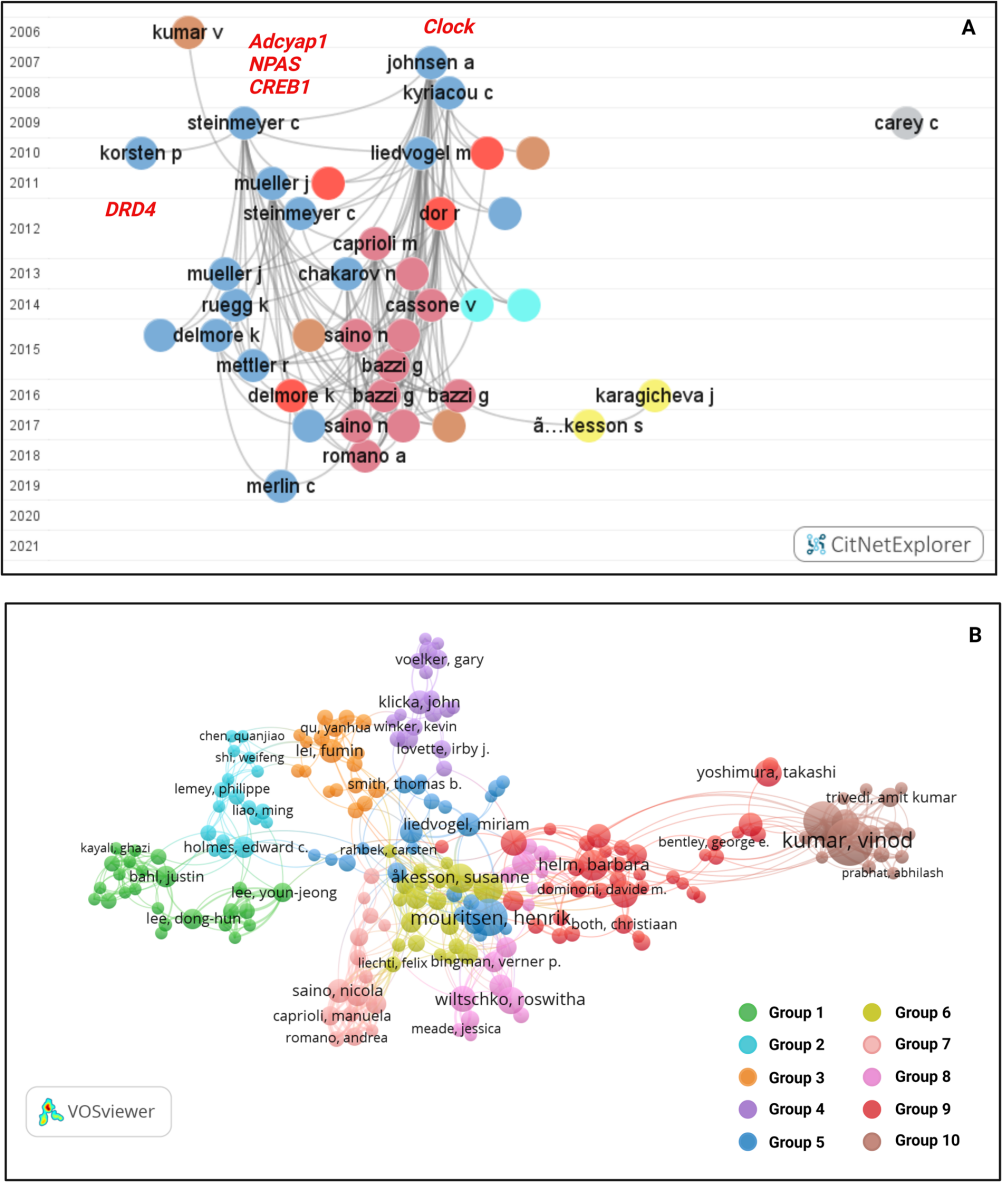
Visualised citation network for studies identified in literature searches. (**A**) Citation network of the Scopus and PubMed database in CitNetExplorer. Publications are organized by year (2006–2021) with the name and first initial of the first author indicating individual studies. The relationship between studies by virtue of co-citations in the reference lists are indicated by grey lines. Subgroup analyses identified several key groups, indicated by the colour code from VOSviewer. Key candidate genes are indicated in red italics and show studies that assayed polymorphisms in the *Clock*, *Adcyap1*, *CREB1*, *NPAS*, and *DRD4* genes. (**B**) Citation network for studies identified in literature searches of the Dimensions and ScienceDirect database in VOSviewer. First authors are labelled by surname and first name. Automated group analyses identified ten clusters of related studies of which the studies identified from Scopus formed part of five groups, indicated as groups 2, 5, 6, 7, 9, and 10. This network shows the larger field of migration studies including non-candidate gene studies such as transcriptomic studies (group 10). (image edited in BioRender.com).

Automated screening for inclusion was done through citation network analyses. For the Scopus database, the results were merged and reformatted with the R package ‘Scopus2CitNet 0.1.0.0’ (https://github.com/MichaelBoireau/Scopus2CitNet) in RStudio 1.4.1106^39^, running R 4.0.5^40^. The results were subsequently visualized by year in CitNetExplorer 1.0.0., keeping only those papers that overlapped in terms of references cited and the largest connected set (Fig. 2a). The results from the search on the Dimensions and ScienceDirect databases were visualized in VOSviewer 1.6.16^35^ by group as well as by year, keeping only those papers that are connected by citations and reference lists (Fig. 2b). The size of bubbles corresponds to citations and the number of cross-links between studies.

### Manual title-abstract screening and full text retrieval

**Fig. 3:**
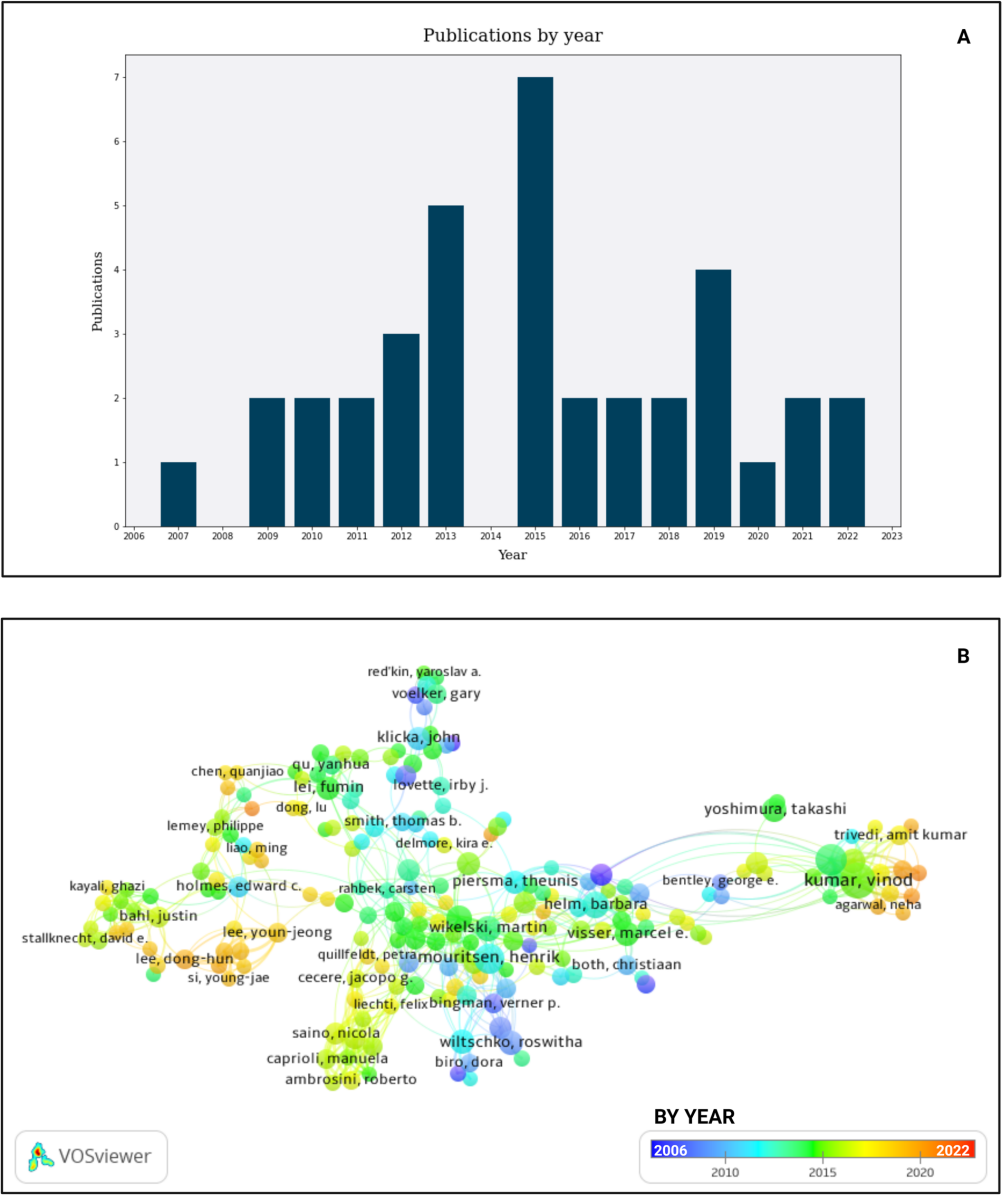
Plots indicating the distribution for publications by year. (**A**) Histogram for publications by year indicating the first publications starting in 2007 up to more recent publications in 2022, with the largest number of publications between 2013–2015 and in 2019. (**B**) Density gradient display of studies in VOSviewer based on year of publication, indicated most studies were published between 2006 (blue) and 2022 (red) with a high number of publications emanating from 2013–2016 (green to orange). (image edited in BioRender.com).

**Fig. 4:**
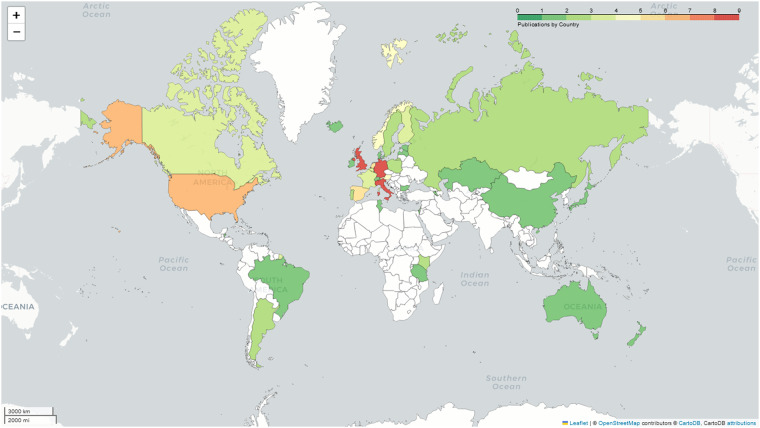
Geographic distribution of candidate gene studies included in the final review dataset (N = 34) based on sampling locations. Related migration studies (N = 32), such as transcriptomic or epigenetic studies, were excluded. The density gradient plots the number of studies per country ranging from one study (green) to more than eight studies (red); countries in white are data deficient. The overall plot indicates that most studies emanated from sampling locations in Europe and North America, with only a small number of studies including sampling from parts of Africa and South America.

Sources identified from the citation networks were imported (citation and abstract) into Mendeley citation manager (www.mendeley.com) for further screening. Several types of studies relating to migration genetics were included in preliminary screening such as candidate gene studies, genomic studies, transcriptomic studies, and epigenetic studies. Studies with a focus on endocrine systems, physiology, or telomeres were excluded. Studies on migration phenology, without an evident genetic link, were also excluded. The inclusion criteria of candidate gene studies were confined to studies that primarily measure *Clock* or *Adcyap1* gene polymorphisms (as well as other candidate genes studied in parallel e.g., *NPAS*, *CREB1*, and *DRD4*: indicated on Fig. 2^34^) within bird populations to compare putative variation to the annual synchronicity in life events and differential migration. These included latitude/longitude/spatial analyses, timing of migration, migratory restlessness, timing of egg laying/breeding, clutch size, moult, urbanisation, and exploratory behaviour. The final set of studies that passed preliminary screening were sought during full text retrieval and added to the imported reference if it wasn’t already included. A total of 66 studies were included in the final review of which 34 were candidate gene studies and 32 were other, migration related, studies using genetic methods. Some basic scientometric assessments of the final set of studies, including the plotting of publications per year (Fig. 3) as well as the geographic distribution (Fig. 4) of studies, was conducted using ABCal version 1.0.2^41^ (https://github.com/LSLeClercq/ABCal).

### Published datasets

A total of 34 studies were identified that used a candidate gene approach for which data retrieval was done. Data was retrieved from either the main text, supplementary material of the article, online data repositories such as Dryad^42–49^ and Figshare^50^, or additional data received directly from authors. Data types varied from allele frequencies to individual level diploid allele data. Allele data for the Barn swallow^51^ was retrieved from the text while data for the Yellow-legged gull^52^ was extracted from images using WebPlotDigitizer version 4.6^53^. Allele data was generally derived from a single source with the exception of the European pied flycatcher^44,49^ and Willow warbler^54–56^. The species, data sources, and data types are summarized in Table 1 along with the sampling location and sample sizes. Frequency data was available for most published studies, with the exception of the bluebird species^18^, and those species for which allele data was unavailable are summarised in Table 2. This includes species for which only frequency data was reported, species for which a non-clock gene approach was used, and studies for which only data summaries without frequencies were reported.

**Table 1:** List of species for which published allele data was collected and/or included in the review and data article.

Common name	Latin binomial	Study	Data	Type	Location	N
Barn swallow*	*Hirundo rustica*	^51,62,63^	^51^	C_A_	Switzerland, Italy	64
Bar-tailed godwit	*Limosa lapponica baueri*	^64^	^64^	C_A_	New Zealand	135
Blackpoll warbler*	*Setophaga striata*	^65^	^43^	C_A_, A_A_	USA	72
Blue tit*	*Cyanistes caeruleus*	^31,32,66,67^	^31,42,45^	C_A_	Europe	950
Collared flycatcher*	*Ficedula albicollis*	^68^	^47^	C_A_, A_A_	Czechia	406
Collared plover	*Charadrius collaris*	^69^	^69^	A_A_	Brazil	14
Common buzzard	*Buteo buteo*	^70^	^48^	A_A_	Germany	978
Common nightingale*	*Luscinia megarhynchos*	^54,71^	^44^, Authors	C_A_, A_A_	Italy	150
Common redstart*	*Phoenicurus phoenicurus*	^54^	Authors	C_A_, A_A_	Italy	43
Common whitethroat*	*Sylvia communis*	^54^	Authors	C_A_, A_A_	Italy	25
Dark-eyed junco*	*Junco hyemalis*	^72^	^72^	C_A_, A_A_	USA	36
Eastern subalpine warbler*	*Curruca cantillans*	^54^	Authors	C_A_, A_A_	Italy	31
Eurasian blackbird*	*Turdus merula*	^22^	^22^	C_A_, A_A_	Europe, Tunisia	792
Eurasian blackcap*	*Sylvia atricapilla*	^73,74^	^50,73^	A_A_	Europe	936
Eurasian golden oriole*	*Oriolus oriolus*	^54^	Authors	C_A_, A_A_	Italy	30
Eurasian hoopoe*	*Upupa epops*	^54^	Authors	C_A_, A_A_	Italy	25
Eurasian reed warbler*	*Acrocephalus scirpaceus*	^54^	Authors	C_A_, A_A_	Italy	24
Eurasian wryneck*	*Jynx torquilla*	^54^	Authors	C_A_, A_A_	Italy	30
European bee-eater*	*Merops apiaster*	^54^	Authors	C_A_, A_A_	Italy	35
European nightjar*	*Caprimulgus europaeus*	^54^	Authors	C_A_, A_A_	Italy	39
European pied flycatcher*	*Ficedula hypoleuca*	^71,75,76^	^44,49^, Authors	C_A_, A_A_	Italy	226
European turtle dove*	*Streptopelia turtur*	^54^	Authors	C_A_, A_A_	Italy	29
Garden warbler*	*Sylvia borin*	^54^	Authors	C_A_, A_A_	Italy	31
Great reed warbler*	*Acrocephalus arundinaceus*	^54^	Authors	C_A_, A_A_	Italy	20
Icterine warbler*	*Hippolais icterina*	^54^	Authors	C_A_, A_A_	Italy	29
Northern wheatear*	*Oenanthe oenanthe*	^54^	Authors	C_A_, A_A_	Italy	30
Painted bunting*	*Passerina ciris*	^77^	^77^	C_A_, A_A_	USA	60
Sedge warbler*	*Acrocephalus schoenobaenus*	^54^	Authors	C_A_, A_A_	Italy	30
Semipalmated plover	*Charadrius semipalmatus*	^69^	^69^	A_A_	Brazil	13
Semipalmated sandpiper	*Calidris pusilla*	^69^	^69^	A_A_	Brazil	14
Spotted flycatcher*	*Muscicapa striata*	^54^	Authors	C_A_, A_A_	Italy	29
Spotted sandpiper	*Actitis macularius*	^69^	^69^	A_A_	Brazil	12
Tree pipit*	*Anthus trivialis*	^54,71^	^44^, Authors	C_A_, A_A_	Italy	153
Tree swallow*	*Tachycineta bicolor*	^16,78^	^46^	C_A_, A_A_	Canada	921
Whinchat*	*Saxicola rubetra*	^54,71^	^44^, Authors	C_A_, A_A_	Italy	208
Willow warbler*	*Phylloscopus trochilus*	^54–56^	Authors	C_A_, A_A_	Italy	495
Wilson’s warbler*	*Cardellina pusilla*	^76^	Authors	C_A_, A_A_	USA	102
Wood warbler*	*Phylloscopus sibilatrix*	^54^	Authors	C_A_, A_A_	Italy	30
Woodchat shrike*	*Lanius senator*	^54^	Authors	C_A_, A_A_	Italy	20
Yellow-legged gull	*Larus michahellis*	^52^	^52^	C_A_, A_A_	Italy	64

Species indicated with an asterisk (*) were included in the allele dataset for population genetics analyses^34^. The primary study, specific data source, location of the study sites and the sample size (N) is given. C_A_: *Clock* gene alleles, A_A_: *Adcyap1* gene alleles.

**Table 2:** List of species for which other published data was collected and/or included in the review and data article.

Common name	Latin binomial	Study	Data	Type	Location	N
African stonechat	*Saxicola torquatus*	^79^	^79^	C_F_	Kenya, Tanzania	172
Asian short-toed lark	*Alaudala cheleensis*	^80^	^80^	C_F_	China	257
Black swan	*Cygnus atratus*	^81^	^81^	Non-CA	Australia	100
Bluethroat	*Luscinia svecica*	^31^	^31^	C_F_	Europe	369
Blue-winged warbler	*Vermivora cyanoptera*	^82^	^82^	Non-CA	USA	24
Canary Island stonechat	*Saxicola dacotiae*	^79^	^79^	C_F_	Canary Islands	61
Chilean swallow	*Tachycineta meyeni*	^16^	^16^	C_F_	Argentina	88
Common buzzard	*Buteo buteo*	^70^	^48^	C_F_	Germany	978
Eurasian blackcap	*Sylvia atricapilla*	^73,74^	^50,73^	C_F_	Europe	936
European roller	*Coracias garrulus*	^14^	^14^	Non-CA	Europe	32
European stonechat	*Saxicola rubicola*	^79^	^79^	C_F_	Europe	382
Golden winged warbler	*Vermivora chrysoptera*	^82^	^82^	Non-CA	USA	42
Great tit	*Parus major*	^83–85^	^83^	C_F_	UK	225
Mangrove swallow	*Tachycineta albilinea*	^16^	^16^	C_F_	Belize	163
Mountain bluebird	*Sialia currucoides*	^18^	^18^	NA	Canada	11
Northern goshawk	*Accipiter gentilis*	^70^	^48^	C_F_	Germany	15
Red kite	*Milvus milvus*	^70^	^48^	C_F_, A_F_	Germany	20
Seychelles warbler	*Acrocephalus sechellensis*	^86^	^86^	Non-CA	Seychelles	57
Siberian stonechat	*Saxicola maurus*	^79^	^79^	C_F_	Kazakhstan, Japan	101
Song sparrow	*Melospiza melodia*	^87^	^87^	Non-CA	Canada	78
Violet-green swallow	*Tachycineta thalassina*	^16^	^16^	C_F_	USA	48
Western bluebird	*Sialia mexicana*	^18^	^18^	NA	Canada	127
White-rumped swallow	*Tachycineta leucorrhoa*	^16^	^16^	C_F_	Argentina	169
Yellow-eyed junco	*Junco phaeonotus*	^72^	^72^	C_F_, A_F_	USA	178

The primary study, specific data source, location of the study sites and the sample size (N) is given. C_F_: *Clock* gene frequencies, A_F_: *Adcyap1* gene frequencies, Non-CA: Non clock gene study, NA: Not Available.

### Unpublished datasets

This study included unpublished data for twelve species in total, summarised in Table 3. The six North American species were sampled at Long Point Old Cut, Ontario, Canada, and included the American redstart (N = 26), Common yellowthroat (N = 31), Hermit thrush (N = 30), Magnolia warbler (N = 33), Swainson’s thrush (N = 29), and White-throated sparrow (N = 32). The six European species included the Common chiffchaff (N = 55) and five species of shearwaters: Barolo shearwater (N = 15), Boyd’s shearwater (N = 25), Great shearwater (N = 25), Manx shearwater (N = 23), and Yelkouan shearwater (N = 15). The Common chiffchaff was sampled from several locations in Sweden (N = 30, subspecies *abietinus*) and Kazakhstan (N = 25, spp. *tristis*). Blood samples were taken from the brachial vein and stored in SET buffer at –80 °C. Shearwaters were sampled from several locations in Europe including France and Portugal while several species were sampled from islands such as Iceland, Cape Verde, and territories of the United Kingdom such as Gough Island. A 1 ml blood sample was taken from the tarsal or the brachial vein during geolocator retrieval. Samples were collected in 1.5 ml plastic tubes containing 70% ethanol and stored at –20 °C until further analysis.

**Table 3:** List of species for which unpublished data was collected and/or included in the review and data article.

Common name	Latin binomial	Study	Data	Type	Location	N
American redstart*	*Setophaga ruticilla*	^34^	Authors	C_A_, A_A_	Canada	26
Barolo shearwater	*Puffinus baroli*	^88^	Authors	C_A_	Portugal	15
Boyd’s shearwater	*Puffinus boydi*	^88^	Authors	C_A_	Cape Verde	25
Common chiffchaff*	*Phylloscopus collybita*	^34^	Authors	C_A_	Sweden, Kazakhstan	55
Common yellowthroat*	*Geothlypis trichas*	^34^	Authors	C_A_, A_A_	Canada	31
Great shearwater	*Ardenna gravis*	^88^	Authors	C_A_	UK	25
Hermit thrush*	*Catharus guttatus*	^34^	Authors	C_A_, A_A_	Canada	30
Magnolia warbler*	*Setophaga magnolia*	^34^	Authors	C_A_, A_A_	Canada	33
Manx shearwater	*Puffinus puffinus*	^88^	Authors	C_A_	Iceland	23
Swainson’s thrush*	*Catharus ustulatus*	^34^	Authors	C_A_, A_A_	Canada	29
White-throated sparrow*	*Zonotrichia albicollis*	^34^	Authors	C_A_, A_A_	Canada	32
Yelkouan shearwater	*Puffinus yelkouan*	^88^	Authors	C_A_	France	15

Species indicated with an asterisk (*) were included in the allele dataset for population genetics analyses^34^. The primary study, specific data source, location of the study sites and the sample size (N) is given. C_A_: *Clock* gene alleles, A_A_: *Adcyap1* gene alleles.

Samples were genotyped using established methods^54^. Briefly, samples of North American species were preserved in a buffer at room temperature until extraction with the ArchivePure DNA purification kit (5 PRIME, Hilden, Germany). Then, polymorphism at *Clock* and *Adcyap1* 3′-UTR was determined as before^54^, with PCR products labelled with HEX (*Clock*), 6-FAM (*Clock* and *Adcyap1*) or TAMRA (*Adcyap1*) dyes. For the Common chiffchaff, genomic DNA was extracted using a standard ammonium acetate protocol. All 55 samples were successfully genotyped and analysed for length polymorphism in the poly-Q repeat of the *Clock* gene following previously published protocols^31^. For Shearwater samples, total genomic DNA was extracted from blood samples using the Speedtools® Tissue DNA Extraction kit (Biotools, Madrid, Spain) following the manufacturer’s instructions. Genotyping was subsequently performed with methods adapted from the literature^31^. Briefly, PCR products were generated with shearwater specific primers for the *Clock* gene labelled with 6-FAM or HEX, followed by fragment analysis as in^54^ to determine the size of the poly-Q repeat.

## Data Records

The data collated during the systematic review and meta-analysis were made available to via the Zenodo repository at the time of publication. Additional inclusion and exclusion criteria were applied and a final set of 40 species (indicated by asterisk in Tables 1, 3) were included in the comparative analyses using mantel and phylogenetic generalised least squares methods to test for an association between migratory phenotypes and candidate gene genotypes^34,57^. This data are available on Zenodo^57^, and includes a workbook with the allele data as well as a results workbook with various population genetics measures including allele frequencies, Homozygosity (H_o_), Heterozygosity (H_e_), Hardy-Weinberg equilibrium^58,59^, and Ewens-Watterson^60^ results. The complete dataset was reformatted for distribution with this data descriptor and is available from two sources, from the Figshare^61^ depository, as submitted with this article, and from a maintained repository with version histories on GitHub^36^.

Data (version 1.0.2) are available as a spreadsheet workbook, labelled “Avian Clock Gene Dataset” with multiple sheets. The first sheet of the workbook, labelled “Index”, contains the table of contents which has several columns (Table 4) that list species by common names, indicates data availability for *Clock* and *Adcyap1*, and total sample size (N). Furthermore, the taxonomic classifications including genus, species, family, superfamily, parvorder, and order are also given. The species codes are hyperlinked to the allele data for individual species, contained in separate sheets within the same workbook. Individual sheets for species contain several columns including the species name, sample ID, and diploid alleles for *Clock* and/or *Adcyap1* genes. Alleles are expressed as the number of polyglutamine repeats (*Q*_*N*_) for *Clock* while the *Adcyap1* alleles represent the amplified fragment length in base pairs (bp). The sum and average of alleles is also provided, and missing data is labelled as NA. For the purpose of individual species analyses, the species sheets from the workbook are also provided as individual comma separated value (CSV) files. The same data is also available on GitHub with the workbooks available in the root directory while the individual CSV files are available in a subfolder with the title “CSV”. The repository also contains a “README” file which provides some basic background and details on the data. Both the workbook as well as CSV files can be read by Microsoft® Office (https://www.office.com/) as well as StarOffice™ (https://www.staroffice.com/), OpenOffice™ (https://www.openoffice.org/), and LibreOffice™ (https://www.libreoffice.org/).

**Table 4:** Description of field names and data for workbook and CSV files.

Field name	Data
*General (Index):*	
Species	Common name in English for species
Clock	Logical binary for data availability of *Clock* gene e.g., “Yes” or “No”
Adcyap1	Logical binary for data availability of *Adcyap1* gene e.g., “Yes” or “No”
Code	Abbreviation used for species tabs
Sample (N)	Size (N) of the total individuals for which data are available
*Taxonomy (Index):*	
Genus	Latin name for genus e.g., “Hirundo”
Species	Latin name for species e.g., “rustica”
Family	Latin name for family e.g., “Hirundinidae”
Superfamily	Latin name for superfamily e.g., “Locustelloidea”
Parvorder	Latin name for parvorder e.g., “Sylviida”
Order	Latin name for order e.g., “Passeriformes”
*Species sheet:*	
Species	Common name in English for species
Sample ID	Sample ID used in raw data for individuals
Clock 1	1^st^ diploid allele for *Clock* gene (individual) as *Q*_*N*_
Clock 2	2^nd^ diploid allele for *Clock* gene (individual) as *Q*_*N*_
Sum	Sum of two alleles for *Clock* gene as *Q*_*N*_
Mean	Mean value of diploid alleles for *Clock* gene as *Q*_*N*_
Adcyap 1	1^st^ diploid allele for *Adcyap1* gene (individual) in base pairs (bp)
Adcyap 2	2^nd^ diploid allele for *Adcyap1* gene (individual) in base pairs (bp)
Sum	Sum of two alleles for *Adcyap1* gene in base pairs (bp)
Mean	Mean value of diploid alleles for *Adcyap1* gene in base pairs (bp)

## Technical Validation

Allele data comprises the heterozygous or homozygous diploid allele for one or both studied clock genes as well as the sum and average of allele sizes. The data for *Clock* was normalized according to the poly-glutamine repeat size (*Q*_*N*_) by subtracting the conserved non-repeat size (*L*_*C*_) in base pairs from the total fragment size (*L*_*T*_) and dividing by codon size, following Eq. 1.

1 $${Q}_{N}=\left({L}_{T}-{L}_{C}\right)/3$$

Data for *Adcyap1* was generated using the same published primers and was kept as the total fragment size.

## Data Availability

The custom R code used to convert data retrieved from Scopus to the appropriate format for visualisation in CitNetExplorer is available from GitHub (https://github.com/MichaelBoireau/Scopus2CitNet). The custom PYTHON script used for plotting the scientometric aspects of the included literature is also available from GitHub (https://github.com/LSLeClercq/ABCal).
