# Author Correction: Birds of a feather flock together: a dataset for ***Clock*** and ***Adcyap1*** genes from migration genetics studies

**DOI:** 10.1038/s41597-024-03352-7

**Published:** 2024-05-13

**Authors:** Louis-Stéphane Le Clercq, Gaia Bazzi, Joan Ferrer Obiol, Jacopo G. Cecere, Luca Gianfranceschi, J. Paul Grobler, Antoinette Kotzé, Marta Riutort León, Jacob González-Solís, Diego Rubolini, Miriam Liedvogel, Desiré Lee Dalton

**Affiliations:** 1 South African National Biodiversity Institute, P.O. Box 754, Pretoria, 0001 South Africa; 2 Department of Genetics, University of the Free State, P.O. Box 339, Bloemfontein, 9300 South Africa; 3 Area Avifauna Migratrice, Istituto Superiore per la Protezione e la Ricerca Ambientale, via Ca’ Fornacetta 9, I-40064 Ozzano Emilia, BO Italy; 4 Departament de Genètica, Universitat de Barcelona, Gran Via de les Corts Catalanes, 585, 08007 Barcelona, Spain; 5 Institut de Recerca de la Biodiversitat (IRBio), Universitat de Barcelona, Gran Via de les Corts Catalanes, 585, 08007 Barcelona, Spain; 6 Dipartimento di Scienze e Politiche Ambientali, Università degli Studi di Milano, via Celoria 26, Milan, I-20133 Italy; 7 Dipartimento di Bioscienze, Università degli Studi di Milano, via Celoria 26, Milan, I-20133 Italy; 8 Departament de Biologia Evolutiva, Universitat de Barcelona, Gran Via de les Corts Catalanes, 585, 08007 Barcelona, Spain; 9 Istituto di Ricerca sulle Acque, IRSA-CNR, Via del Mulino 19, I-20861 Brugherio, (MB) Italy; 10 Max Planck Research Group Behavioural Genomics, Max Planck Institute for Evolutionary Biology, 24306 Plön, Germany; 11 Institute of Avian Research, An der Vogelwarte 21, 26386 Wilhelmshaven, Germany; 12 School of Health and Life Sciences, Teesside University, Middlesbrough, TS1 3BA UK

**Keywords:** Behavioural genetics, Animal migration, Animal migration, Behavioural genetics, Animal migration, Animal migration

Correction to: *Scientific Data* 10.1038/s41597-023-02717-8, published online 09 November 2023

In the Acknowledgement section of this article, the following grant numbers were omitted:

- Grant number 20178T2PSW from the PRIN 2017 for Diego Rubolini;
- Grant number PID2021-125792NB-I00 from the Ministerio de Ciencia e Innovación, Agencia Estatal de Investigación to Marta Riutort

In addition, the ICREA Academia award to Jacob González-Solís was omitted.

The original article has been corrected.

